# Gas Pressure From the Endoscope: An Unexplored Contributor to Morbidity and Mortality?

**DOI:** 10.7759/cureus.31779

**Published:** 2022-11-22

**Authors:** Richa Wardhan, Nikolaus Gravenstein, Peter Draganov, Alberto Bursian, Jeffrey D White

**Affiliations:** 1 Anesthesiology, University of Florida, Gainesville, USA; 2 Medicine, University of Florida, Gainesville, USA

**Keywords:** endoscopy ercp, nora, covid, gas embolism, insufflation pressure, venous air embolism, gastric endoscope

## Abstract

Background

It has been shown that the incidence of venous air embolism and venous carbon dioxide (CO_2_) embolism is high during endoscopic retrograde cholangiopancreatography (ERCP). We examined insufflating gas flow and maximum pressure produced by three types of commonly used endoscopes because we could not readily locate technical data for endoscope gas flow and maximum emitted pressure in the manufacturer's manuals.

Methods

We tested the Olympus GIF-Q180 used for esophagogastroduodenoscopy, the CF-Q180 used for colonoscopy, and the TJF-Q180 used for ERCP (Olympus America Inc., Center Valley, Pennsylvania). Under three different clinical gas insufflation scenarios, we measured in vitro maximum gas pressure transduced from a closed space created at the endoscope tip in a worst-case scenario analysis.

Results

We showed that it is readily possible to generate a pressure (>5-30 times normal central venous pressure) in the air space at the tip of all three endoscopes when insufflation is activated and the gas egress is limited.

Conclusions

These findings shed additional light on in vivo occurrences of gas embolism during gastrointestinal endoscopy. We postulate that in addition to using exclusively CO_2_ as the insufflating gas, the risk of gas embolism can be further diminished by regulating insufflating gas pressure at the tip of endoscopes.

## Introduction

Gastrointestinal (GI) endoscopy is a very commonly performed, low-morbidity procedure [1-5]. The overall rate of cardiopulmonary adverse events ranges from 1 in 170 to 1 in 10,000 [6]. In the United States, GI endoscopy is most performed in a dedicated outpatient endoscopy center or an in-hospital non-operating room setting. Death during this procedure is rare. Due to the largely elective nature of GI endoscopy procedures, any significant morbidity or fatality comes as a devastating, unexpected outcome for the patient, the patient's family, and the endoscopy team.

One of the significant reported causes of morbidity/fatality associated with GI endoscopy is venous gas embolism (VGE) [7]. The risk for gas embolism during endoscopy appears highest during endoscopic retrograde cholangiopancreatography (ERCP) [3,8-10]. Less commonly, gas embolism has also been reported during esophagogastroduodenoscopy (EGD), colonoscopy, and even sigmoidoscopy [11-13]. Besides the risk of embolism, iatrogenic colonoscopic perforations can infrequently result from excessive air insufflation or barotrauma, amongst other etiologies [14].

Logically, the source of the gas embolism and bowel perforation is the insufflating gas and associated gas pressure used to distend the lumen and enable the advancement of the endoscope into the area being examined or treated. We could not find technical data in the manufacturer's manuals for the Olympus (Olympus America Inc., Center Valley, Pennsylvania) endoscope gas flows and emitted pressure for the endoscopes commonly used at our facility. Therefore, we assessed gas flow and gas pressure beyond the tip of the endoscopes using an in vitro model.

## Materials and methods

These experiments were exempt from the University of Florida Institutional Review Board approval.

Endoscopes and processors

We tested the Olympus GIF-Q180 used for EGD, the Olympus CF-Q180 used for colonoscopy, and the Olympus TJF-Q180 used for ERCP (Olympus America Inc., Center Valley, Pennsylvania). Each of the endoscopes was connected to an Olympus Evis Exera III CLV-190 unit, which provides a light source. The Evis Exera III CLV-190 unit has the capability to provide insufflating gas flow regulation and was explicitly designed to use air as the insufflating gas. The CLV-190 gas flow regulation function can also be bypassed (designated below as "bypass") [15]. If the CLV-190 unit is used for insufflation gas flow regulation, it can be set to high (the default), medium, or low. Olympus manufactures a separate insufflation gas regulator called the UCR CO2 Regulation Unit specifically for the delivery of carbon dioxide (CO2) as the insufflating gas [15]. Like the CLV-190, the UCR CO2 has three gas flow settings: high (the default), medium, or low. To achieve the three different CO2 gas flow settings with the UCR CO2, it should be connected to the endoscope with a special proprietary gas tubing.

In vitro system setup

We measured the maximum pressure and flow at the distal end of the endoscope in three configurations.

For the first set of measurements, our GI technicians prepared each of the three endoscopes in the manner our center currently uses (bypass) for endoscopic examination: the endoscope insufflating gas supply is connected to a wall source CO2 flowmeter set at 2 L/min. This is connected to the endoscope air supply connector. In this setup, the gas dispensing function of the Olympus Evis Exera III CLV-190 processor is bypassed, and the flow of gas through and out of the endoscope is primarily determined by the setting of the wall source CO2 flowmeter. 

The CO2 source was connected to the Olympus Evis Exera III CLV-190 unit for the second set of measurements. Maximum pressure at the end of the endoscope was determined at high- and low-flow settings. 

The CO2 source was connected to the Olympus UCR CO2 gas regulator for the third set of measurements. Maximum pressure at the end of the endoscope was determined at high- and low-flow settings. We used the proprietary UCR CO2 high- and low-flow gas tubing to achieve the manufacturer's intended gas flow rate.

Measurements

**Figure 1:**
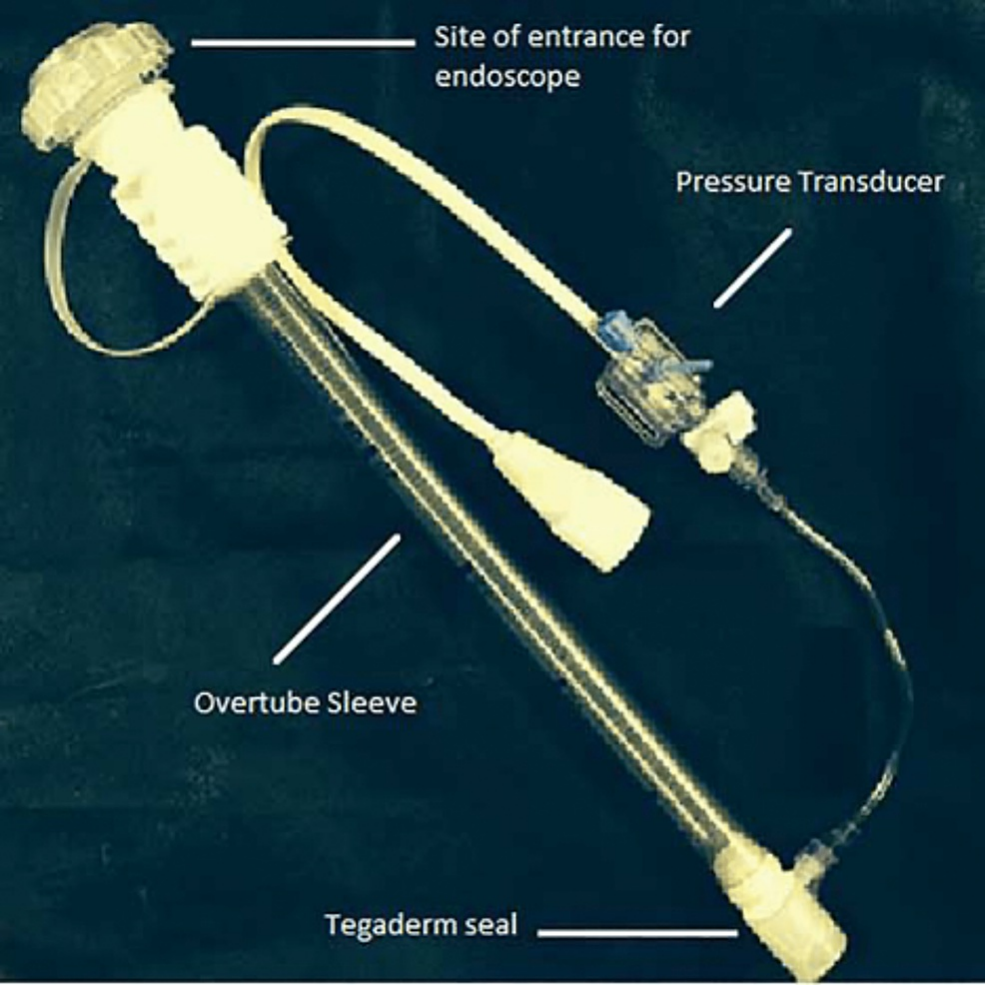
Setup for measurement of endoscope tip pressures

**Figure 2:**
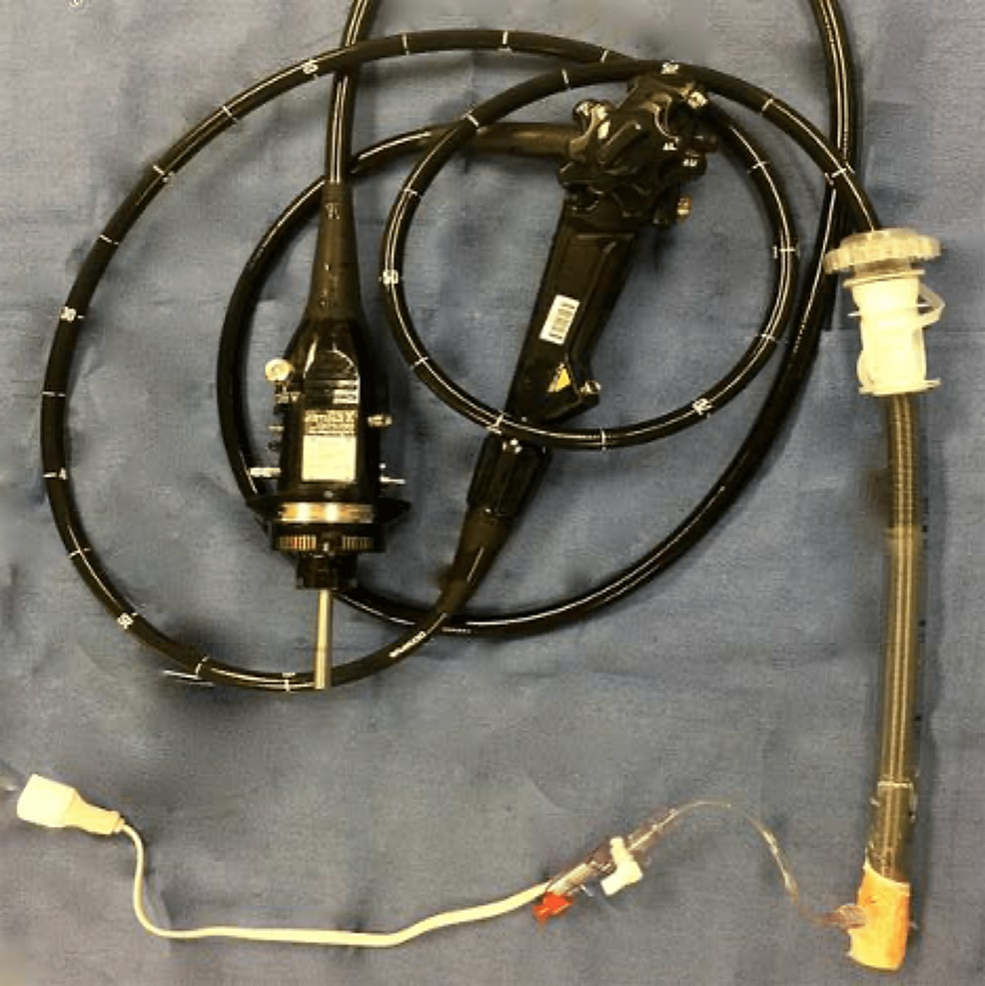
Endoscope seated within Overtube setup for measurement of tip pressure

To measure gas pressure at the exit port of the endoscope, we used a plastic US Endoscopy Guardus® Overtube (US Endoscopy, Mentor, Ohio) with a Tegaderm® (3M United States, St. Paul, Minnesota) seal at the distal end. A distal side port connected to an arterial pressure transducer (Edwards Lifesciences Corp, Irvine, Califonia), as shown in Figure 1. We seated each endoscope within the Overtube sleeve to ensure a relatively complete seal for both ends (Figure 2). We then depressed the gas flush button and measured gas pressure within the Overtube sleeve six times for each endoscope. The Overtube was depressurized between trials.

We set the wall source CO2 flowmeter at 2 L/min to measure flow rates. We then fed the distal end of each endoscope just within the mouth of an inverted full 1-L bottle of water within a water-filled basin to keep the bottle sealed. Finally, we fully depressed the endoscope gas flush button and measured the time to displace the liter of water six times for each endoscope.

## Results

The measured flow under the bypass setup for each of the three different endoscopes was the same. There was good agreement between the set gas flow at the wall source and the measured gas flow at the endoscope tips. All conditions resulted in pressure within the sealed Overtube that was too high to calculate with our transducer (i.e., >300 mmHg), as shown in Table 1.

**Table 1:** Results for Olympus endoscopes set up to bypass the Evis Exera III CLV-190 The CO2 gas source was the wall source CO2 with the flowmeter set at 2 L/min.

Endoscope	Gas flow (L/min)	Pressure (mmHg)
GIF-Q180	1.8	>300
CF-Q180	1.8	>300
TJF-Q180	1.8	>300

Our findings are similar to the technical specifications data released by the Technology Assessment Committee of the American Society for Gastrointestinal Endoscopy (ASGE), which reports approximately 180 mm Hg emitted at the tip of the endoscope using air or CO2 [14].

The measured flow under the bypass setup for each of the three different endoscopes was the same. There was good agreement between the set gas flow at the wall source and the measured gas flow at the endoscope tips. All conditions resulted in a pressure within the sealed Overtube that was too high to measure with our transducer (i.e., >300 mmHg), as shown in Table 1.

The measured data from the UCR CO2, according to the ASGE listed in Table 2, appears to agree with our flow data from the CO2 bypass setup [14,16].

**Table 2:** UCR CO2 flow data according to the American Society for Gastrointestinal Endoscopy

UCR-CO_2_	Flow (L/min)
Low flow	1.2
High flow	1.8

When plumbed through the Evis Exera III CLV-190 and the UCR CO2 regulator, the three endoscopes generated endoscope tip pressures that were lower than the bypass setup. In the case of the EGD and ERCP scopes, these pressures were still significantly higher than normal central venous pressure (<10 mm Hg). Even with the colonoscope, the tip pressures generated via the Evis Exera III CLV-190 and UCR CO2, though lower than the wall gas flow, still reached many times the normal central venous pressure (Table 3). 

**Table 3:** Results for three endoscopes set up with gas supply from Olympus UCR CO2 regulator or Olympus Evis Exera III CLV-190

Scope	UCR setting low flow (mean mmHg + SD)	UCR setting high flow (mean max mmHg + SD)	Exera setting low flow (mean max mmHg + SD)	Exera setting high flow (mean max mmHg + SD)
EGD-GIF-Q180	165.8 ± 7.6	207.8 ± 11.3	126.2 ± 13.0	144.2 ± 5.2
Colonoscope CF-Q180	44 ± 4.6	85.6 ± 3.2	96.2 ± 1.9	119.5 ± 2.6
ERCP-TJF-Q180	175.5 ± 5.3	192.7 ± .5	161.3 ± 4.2	198.5 ± 7.8

## Discussion

The American Society of Anesthesiologists Closed Claims database compares the percentage of malpractice claims for death and hypoxic injury for surgeries performed in non-operating room anesthesia (NORA) locations versus those done in an operating room suite [17,18]. NORA claims for death and hypoxic injury are 1.8 and seven times the number of operating room claims, respectively. 

Along with the heightened awareness of perioperative risks in the NORA arena, the possibility of gas embolism during endoscopy should be kept in mind in all GI endoscopy cases and, in particular, ERCPs, even when CO2 is substituted for air as the insufflation gas medium [8,9]. Gas embolism is mainly associated with ERCP, but it has also been reported after other GI endoscopy procedures.

As currently manufactured, the only way to somewhat regulate the otherwise unregulated endoscope tip pressure is via the insufflating gas flow setting. This can be achieved by setting the flow rate at the wall gas source, commonly at 2 L/min, if no intermediate regulator machine is used (the bypass setup); by setting the flow at high, medium, or low on the Evis Exera III CLV-190; or by using special tubing (high, medium, low) and the corresponding applicable flow setting on the UCR CO2. 

Under all setup conditions examined, if the EGD, ERCP, or colonoscope tip is positioned near tissue where blood vessels are open and in continuity with the gas, the potential intravascular gas infusion rate can easily exceed 300 mL/min. In canine models of gas embolism, this rate exceeds the capacity of the pulmonary microcirculation to absorb gas bubbles, thus creating venous to the arterial transmission of gas independent of an anatomical shunt [19]. Under the bypass setup, a 2 L/min wall source setting can readily produce a critical gas embolism. Screening colonoscopy procedures are typically reported to use a mean of 8 L of insufflating gas (air or CO2) [14,20], with time-averaged insufflating flow rates that range from 30 to 950 mL/min. Such gas volumes and flow rates have been postulated to create deleterious physiologic changes leading to cardiopulmonary collapse via two nonembolic tension pneumoperitoneum mechanisms: hyperacute intraluminal viscus dilation and extraluminal abdominal compartment syndrome [21] following unrecognized viscus perforation by the endoscope tip.

Under all three tested Olympus endoscope setup conditions, if an EGD, ERCP, or colonoscope tip is near an open blood vessel, the insufflating gas is emitted at a pressure that always has the potential to be much higher than local venous or even arterial or arteriolar pressure if it is not decompressed or vented or if it leaks out of the structure being inspected or treated. If the insufflating gas is used partly to improve visualization from local oozing, the risk of intravascular gas embolization becomes even more significant.

Endoscopy practitioners should have a heightened awareness of the possible ill effects of performing endoscopy with large volumes of insufflating gas under potentially high local pressure near the endoscope tip, especially when the insufflating gas is air. GI endoscopy is well represented in the American Society of Anesthesiologists Closed Claims Database [22]. Thus, it is equally crucial for anesthesiologists and endoscopists to be aware of the signs, symptoms, and management of gas embolism. For additional information on this topic, we recommend an excellent infographic by Wanderer and Nathan [9].

Because of its favorable solubility properties and ready availability, we recommend that CO2 should be replaced with air as the insufflating medium of choice and that gas regulating units should be preferred over endoscope setups that bypass them whenever possible to provide better control of insufflating gas flows and pressures.

Regarding strengths and limitations, we believe that the in vitro model we used for measuring endoscope tip gas pressure is a reasonable proxy for a worst-case scenario of an in vivo lumen. Our model provided measurements in the range of those reported in an ASGE technical data document [14]. Our measures provide a logical explanation and mental model for in vivo, endoscopy-related, clinically significant gas embolism events. The implications of our findings have not been widely published, except in several gastrointestinal medicine publications [14,23], such as the ASGE Technology Assessment Committee document. In the anesthesiology literature, the prospective study by Afreen et al. [9,23] established that the venous air embolism incidence could be 2% (for air).

In contrast, venous CO2 embolism rates could be 4% (for CO2) [23] during ERCP. Our study helps to fill in the lack of readily available technical data for Olympus endoscope gas flows and emitted tip pressures, as they are not currently listed in the manufacturer's manuals [23]. 

Limitations

We recognize that our model does not reproduce the actual physiologic conditions of an intestinal lumen. The plastic Guardus® Overtube sleeve does not share the distensibility and compliance characteristics of a bowel or bile duct segment. 

Another potential weakness of our prototype is that we sealed one end of the Overtube to provide a relatively closed system and created only a worst-case scenario to measure the potential for high gas pressure beyond the tip of the endoscope. In a typical clinical setting, there would generally be no such physical barrier to gas flow exit and the offset of high gas pressures by gas displacement down the intestinal lumen. Our in vitro model may apply to anatomical intestinal blockage (i.e., post-surgical stricture, bowel obstruction, diverticulum) or a functional defect (i.e., abnormal or absent peristalsis) [21]. Regardless, the only reason a lumen is ever distended larger than the diameter of the endoscope during examination is the gas pressure in the lumen. To better define the implications of this in vitro study, intraluminal pressures should be assessed in cadavers and patients.

## Conclusions

The insufflating gas pressure through three standard Olympus GI endoscopes is not pressure-limited, even when using proprietary insufflating gas regulator devices.

We recommend that Olympus modify the default flow setting for the Evis Exera III CLV-190 and the UCR CO2 from high (as it is currently) to low. We would also strongly encourage a mandate for the widespread and exclusive use of CO2 rather than air as the endoscope insufflating gas of choice because of its far more favorable blood solubility (approximately 35-50 times that of nitrogen) should the distending gas gain entrance to the vascular system.

Our albeit ingenious model indicates that even at a low setting, the Olympus CLV-190 and UCR CO2 can still produce in vitro luminal pressures that vastly exceed in vivo normal central venous pressure. Given the high fatality rate among 29 published case reports for ERCP-related air embolism (with 14 fatalities reported), we have an opportunity and worthy goal to reduce gas embolism during endoscopy.
